# A Preliminary Computational Investigation Into the Flow of PEG in Rat Myocardial Tissue for Regenerative Therapy

**DOI:** 10.3389/fcvm.2019.00104

**Published:** 2019-08-07

**Authors:** Malebogo Ngoepe, Andreas Passos, Stavroula Balabani, Jesse King, Anastasia Lynn, Jasanth Moodley, Liam Swanson, Deon Bezuidenhout, Neil H. Davies, Thomas Franz

**Affiliations:** ^1^Department of Mechanical Engineering, University of Cape Town, Rondebosch, South Africa; ^2^Wallenberg Research Centre, Stellenbosch Institute of Advanced Study, Stellenbosch University, Stellenbosch, South Africa; ^3^Department of Mechanical Engineering, University College London, London, United Kingdom; ^4^Cardiovascular Research Unit, Department of Surgery, University of Cape Town, Observatory, South Africa; ^5^Division of Biomedical Engineering, Department of Human Biology, University of Cape Town, Observatory, South Africa; ^6^Bioengineering Science Research Group, Engineering Sciences, Faculty of Engineering and the Environment, University of Southampton, Southampton, United Kingdom

**Keywords:** myocardial infarction, injectate therapy, polyethylene glycol hydrogel, particle image velocimetry, computational fluid dynamics, retention

## Abstract

Myocardial infarction (MI), a type of cardiovascular disease, affects a significant proportion of people around the world. Traditionally, non-communicable chronic diseases were largely associated with aging populations in higher income countries. It is now evident that low- to middle-income countries are also affected and in these settings, younger individuals are at high risk. Currently, interventions for MI prolong the time to heart failure. Regenerative medicine and stem cell therapy have the potential to mitigate the effects of MI and to significantly improve the quality of life for patients. The main drawback with these therapies is that many of the injected cells are lost due to the vigorous motion of the heart. Great effort has been directed toward the development of scaffolds which can be injected alongside stem cells, in an attempt to improve retention and cell engraftment. In some cases, the scaffold alone has been seen to improve heart function. This study focuses on a synthetic polyethylene glycol (PEG) based hydrogel which is injected into the heart to improve left ventricular function following MI. Many studies in literature characterize PEG as a Newtonian fluid within a specified shear rate range, on the macroscale. The aim of the study is to characterize the flow of a 20 kDa PEG on the microscale, where the behavior is likely to deviate from macroscale flow patterns. Micro particle image velocimetry (μPIV) is used to observe flow behavior in microchannels, representing the gaps in myocardial tissue. The fluid exhibits non-Newtonian, shear-thinning behavior at this scale. Idealized two-dimensional computational fluid dynamics (CFD) models of PEG flow in microchannels are then developed and validated using the μPIV study. The validated computational model is applied to a realistic, microscopy-derived myocardial tissue model. From the realistic tissue reconstruction, it is evident that the myocardial flow region plays an important role in the distribution of PEG, and therefore, in the retention of material.

## Introduction

The prevalence of non-communicable diseases has risen significantly in the last few decades, and this has affected societies in a myriad of ways. In low- and middle-income countries, cardiovascular and other non-communicable diseases tend to affect younger people who are economically active, and this has a negative impact on the general well-being of these nations (1, 2). Recognition of these changing trends has led to various shifts in global health policy (3, 4). From a healthcare management perspective, greater focus has been placed on prevention of lifestyle diseases, but also on improved understanding of disease progression. This has created an impetus for the development of appropriate solutions for long term management or mitigation of disease. In the last decade, regenerative medicine has gained a lot of attention as a potential long-term solution for a broad range of diseases. Included in these conditions of interest is myocardial infarction (MI), more commonly known as heart attack (5).

The end result of MI is the loss of a large number of cardiomyocytes and deposition of scar tissue at the site of injury. This leads to a decrease in left ventricular function, placing strain on the rest of the heart to keep supplying the body with adequate volumes of blood at the correct rate. Existing treatments for myocardial infarction focus on limiting the amount of damage that the tissue suffers but ultimately, they just serve to prolong the time to heart failure (6, 7). While it is highly unlikely that the injured myocardium can ever return to its original state, some functional benefit has been gained from injecting stem cells into the injured part of the heart (8). The exact mechanism of healing following stem cell engraftment is still unclear. Some studies have shown that the paracrine effects of stem cell associated growth factors contribute significantly to healing (9–12).

The main challenge with the efficacy of stem cell treatment in myocardial infarction, in particular, is the low retention of cells following injection (13). Owing to the vigorous action of the heart, a number of injected cells are transported via the systemic circulation to other organs of the body, while others are ejected via the needle track (14–16). Factors such as delivery route and injection timing have been investigated as possible contributors to retention (17–22). Even with the massive losses of injected cells, improved left ventricular function has been observed. This has encouraged development of scaffolds and biomaterials which can be injected with the stem cells, aiding the engraftment process. In some instances, the biomaterials have been seen to improve ventricular function without the stem cells, leading to numerous studies attempting to optimize their properties (23). Hydrogels are a type of scaffold that has been widely explored for these purposes and can be derived from natural or synthetic materials.

Polyethylene glycol (PEG) gel and its modified forms have been used as an injectate for myocardial infarction therapy development (24–28). Synthetic PEG gels can be readily customized and optimized for specific purposes. When a suitably functionalised PEG is prepared in solution and combined with a cross-linker, it can be injected into the heart in a fluid state and then spontaneously gelled over time, *in situ*. A key observation that has been made about the gel following injection is that it distributes itself differently in the myocardial tissue, depending on the injection timing (24). Less gel is retained if injection happens immediately after infarction while delayed delivery results in improved retention and functional benefit. Such observations have led to a greater desire to understand the mechanism of PEG distribution and retention once injected.

To this end, a number of computational models have been developed in an effort to understand the behavior and effect of injectable scaffolds (29–37). Most of these models have focused on biomaterials in their gelled state and have examined how the presence of the gel in the myocardial tissue affects stress response in the wall. Given that the greatest losses are likely to occur before the gelled state is reached, interest has been expressed in the behavior of the gel while still in a liquid state. While there are currently no *in silico* studies examining the behavior of liquid PEG in myocardial tissue, there are a vast number of experimental studies that have examined the behavior of PEG in a wide variety of circumstances. These often consider the modified forms of PEG or the combined response of the gel and stem cells (38–41). The main challenge is that only a very small subset of these studies focus on PEG in its pre-altered form, hence making it difficult to state its fundamental rheological behavior. A number of studies focusing on the PEG monomer have examined how variables such as pH, molecular weight, and temperature alter rheological behavior (42–48).

For most of the studies focusing solely on PEG, the material has been shown to have constant viscosity within a specified shear rate range, and this value varies according to the molecular weight of the gel and the testing conditions. Dontula et al. used PEG 8000 as a reference in a study examining the behavior of PEG solutions (42). The reference solution exhibited Newtonian behavior up to a shear rate of 100 s^−1^. In a study that examined the effect of thermal history on the behavior of PEG (3, 6, and 10 kDa), Baldursdottir et al. found that viscosity decreased dramatically within the first 1°C of temperature change and then stabilized at a constant value for each different molecular weight (44). The experiments were all conducted at the same shear rate (0.1 s^−1^), hence it was not possible to conclude how the viscosity would change with shear rate. It was evident, however, that following the initial drop in temperature, viscosity generally remained constant. Azri et al. characterized the linear viscoelastic behavior of low molecular weight PEG by studying the rheological response of 0.6 kDa PEG over a frequency range of 0.16–16 Hz (46). The experiments were conducted at different temperatures and it was found that all samples exhibited Newtonian behavior over the entire range of frequencies. In a study using 4 kDa PEG, Brikov et al. also found that viscosity is constant until 1,000 s^−1^ (47). Contrary to this general trend, Liu et al. found that PEG was shear-thinning over a shear rate range of 2,000 s^−1^ (48). In their study, they considered the rheological character of PEG over a range of molecular weights (0.4, 0.6, 1, 6, 10, and 20 kDa). For all the samples, viscosity was seen to decrease as shear rate increased.

The wide range of testing conditions and methods employed for characterizing the rheological behavior of PEG make it difficult to draw exact conclusions. For example, some of the studies make use of a viscometer, which produces information about viscosity, but not about the overall flow behavior of a fluid (43, 45, 49). In addition, available literature on PEG tends to focus on a particular molecular weight or temperature, which is of interest for further studies. The assumption that the relative simplicity of the material would have resulted in complete characterization does not seem hold, and the lack of standard testing conditions (e.g., concentrations, temperatures, and pH) makes it difficult to compare results. Furthermore, most of the rheological techniques in these studies characterize fluid behavior on the macroscale. This may result in incorrect characterization of dynamics that arise on the microscale (50–52). This is particularly true in the case of polymers, which experience restrictions on conformation in near-wall regions and behave slightly differently in microflows (53, 54).

In this study, we aim to characterize and study the flow behavior of a 20 kDa PEG solution in myocardial tissue, after injection but prior to gelation, when the greatest losses are likely to occur. The vast majority of results presented from rheological tests, in literature, state that PEG behaves as a Newtonian fluid within a specific shear rate range. Its microscale flow behavior, where viscous forces are likely to be dominant, is unknown and is an important factor to consider when modeling flows in myocardial tissue. Micro particle image velocimetry (μPIV) was used to observe the flow profiles of PEG solution in a microchannel, which represents the gaps in myocardial tissue, and is comparable in size. Even though extensive remodeling takes place within the myocardial tissue following infarction, the early stage post-infarct, which is considered in this model, is adequately represented by this microchannel. The data obtained from these μPIV experiments was used to characterize the behavior of the fluid on the microscale. An idealized two-dimensional computational fluid dynamics (CFD) model, replicating the experimental microchannel, was then developed and validated using the μPIV data. Even though the geometry in question is fairly simple, validation is important as there are no analytical solutions for the flow of non-Newtonian fluids in non-circular geometries. The idealized CFD model was then extended to a three-dimensional model of realistic myocardial tissue derived from confocal microscopy. The myocardial tissue resembles normal tissue, and, like the microchannel, reflects the early stage post-infarct. As a proof of concept, this model was used to investigate whether or not the myocardial flow region had a significant impact on gel transport.

## Materials and Methods

The study comprised two main parts. The *in vitro* study sought to establish the flow behavior of PEG solution in microchannels, using μPIV. The *in silico* study, which used some of the data from the *in vitro* study, then examined flow in idealized and realistic geometries.

### *In vitro* Study

In order to characterize the flow of PEG in microchannels representing the gaps in myocardial tissue, a solution of PEG had to be prepared. This was then perfused through a microchannel, and the flow was imaged and quantified using a μPIV setup. Finally, a set of equations was used to characterize flow behavior and extract key parameters. Details of each stage are given below.

### Polyethylene Glycol Solution Preparation

PEG solution was prepared using a protocol adapted from the work of Kadner et al. (24). A 20 kDa, 8-arm hydroxyl-terminated PEG (20PEG-8OH, Nektar) was used for the study. A 10% m/v solution of PEG was prepared by dissolving 1 g of powdered PEG-OH in 10 ml PBS. Unlike other injectate studies where a cross-linker is added for gelation, the solution was left as is to enable observations in the liquid state, when the greatest losses are most likely to occur *in vivo*.

### μPIV Study

**Figure 1:**
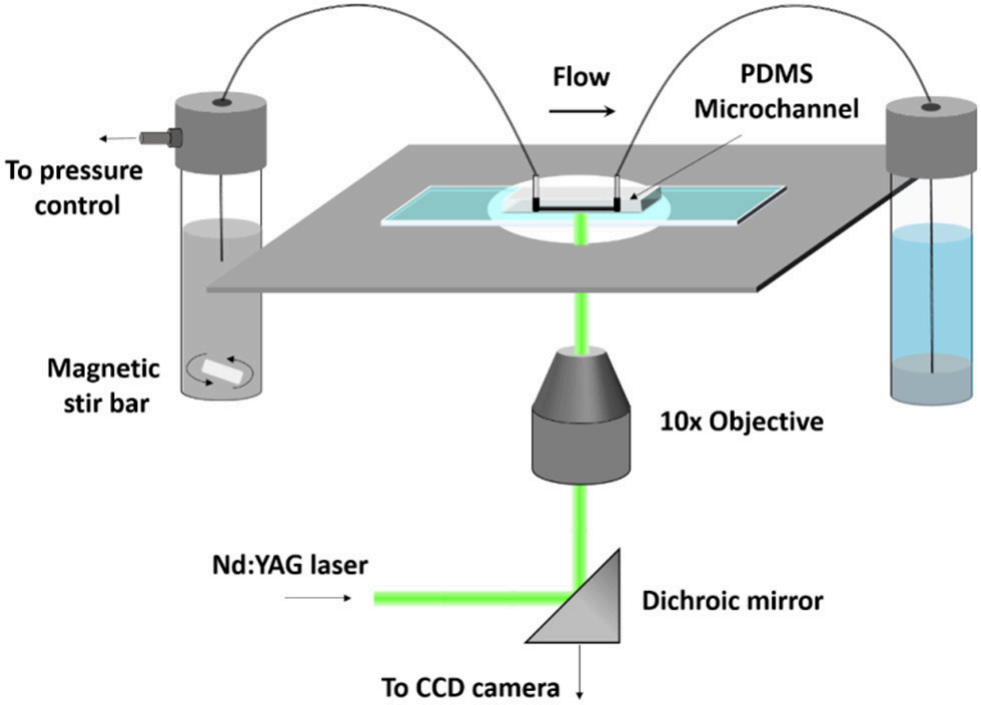
Schematic of the μPIV system used in this study.

The flow of the solution was observed in a polydimethylsiloxane (PDMS) microchannel with a square cross-section of 50 × 50 μm and length of 15 mm. The dimensions of the microchannel approximated the gaps through which the solution would flow in myocardial tissue. The flow rate of the solutions was regulated using a custom made pneumatic microfluidic flow controller as described in Sherwood et al. (55). The microchannel was placed on an inverted microscope (DMILM: Leica). The flow was seeded with 1 μm density-neutral fluorescent microspheres (Nilon Red, Invitrogen) and illuminated by means of a pulsed Nd:YAG laser (wavelength 532 nm, New Wave, USA). The focal plane was position at the central plane of the channel and 60 pairs of images were acquired by a CCD camera (Hamamatsu, UK:C8484-05C) with 10x magnification and sampling rate of 6 Hz. The time interval between the pairs of images was 0.5 ms. Image acquisition was controlled via LabVIEW (National Instruments) and images were pre-processed with MATLAB. A schematic of the μPIV system is illustrated in Figure 1. The velocity was obtained from the acquired images using multipass PIV cross-correlation algorithms implemented in open source software JPIV. Three pass ensemble average correlation was utilized with interrogation windows of 8 pixels height and widths of 32, 16, and 16 pixels, respectively, with 50% overlap resulting in a vector field with a spatial resolution of 2.4 μm across the flow. A region of interest (ROI) three channel widths long and located at the middle of the microchannel, i.e., 7.5 mm downstream of the inlet, was selected to estimate the velocity profiles across the channel. The distance from the wall to the first measurement point was 2 μm. The nominal shear rate, defined as the ratio of the average velocity to channel width, ranged from 8 to 132 s^−1^ in this study. The average velocity (and the flowrate) was determined by integrating the measured velocity profiles. The estimated flow rates (ranging from 2.19 × 10^−8^ to 3.42 × 10^−7^ m^3^s^−1^) were used as input in the CFD simulations for the simplified channel geometry. Thus, any effects due to depth of correlation (equal to 36 μm in this set up) will be minimized or cancel out when making experimental and CFD comparisons.

### Characterizing Fluid Behavior From μPIV Velocity Profiles

The data obtained from the μPIV study was analyzed and fitted using equations described in the paragraphs that follow. From this data, the rheological behavior of the fluid was deduced. Details of the steps are given in the paragraphs that follow.

The measured velocities across the microchannel were fitted to an experimental data curve derived by Sherwood et al. using MATLAB's non-linear fitting function (55). This experimental curve corrects for irregularities and asymmetries which are present in the measured data. The curve used for fitting is given by Equation (1),

(1) $$\begin{array}{r} v_{experimental}=v\left(\frac{\cosh\left(0.5^{m}\right)-\cosh\left(|y_{i}^{*}{|}^{m}\right)}{\cosh\left(0.5^{m}\right)-1}\right)+v_{0} \end{array}$$

where v_experimental_ is velocity for the experimental curve, v is a weighting parameter related to average velocity, m is an exponent that describes the bluntness of the velocity profile, ${\text{y}}_{\text{i}}^{*}$ is the y co-ordinate normalized by width (y/w) and ranges from −0.5 to 0.5, and v_0_ is the wall slip velocity. Wall slip was included in the fitting as PIV readings could not be accurately obtained near the wall; hence the first velocity measurement was taken 4 pixels from the wall. Furthermore, it is important to consider wall slip in polymer solutions, which have features that deviate from the no-slip condition. The slip layer, which is influenced by polymer concentration and particle size, can be >2 μm (53). Often, the Equation (1) is a simplification of the analytical velocity profile for laminar Newtonian flow in rectangular channels (56). The two-dimensional analytical velocity profile is given in Equation (2),

(2) $$\begin{array}{r} v_{analytical}=\;\frac{4h^{2}\Delta p}{\pi^{3}\mu L}\;\overset{\infty }{\underset{n,odd}{\sum }}\frac{1}{n^{3}}\left[1-\;\frac{cosh\;\left(n\pi \frac{y}{h}\right)}{cosh\;\left(n\pi \frac{w}{2h}\right)}\right] \end{array}$$

where v_analytical_ is the analytical velocity for a given y position, h is the height of the channel, Δp is the pressure gradient across the channel length, μ is the dynamic viscosity, L is the length of the channel, and w is the channel width. The deviation of the measured velocity profiles from the analytical solution provides a measure of the extent of shear thinning of the fluid as the latter tend to produce blunter profiles.

Once the experimental curves were obtained, they were normalized with the average velocity so that the power law index for each case could be retrieved. It is important to note that there is no analytical solution for the flow of a power law fluid in a rectangular duct (52). Fortunately, the power law index is independent of the cross-sectional shape of the duct (57). For this reason, the power law index can be calculated using the analytical solution for a power law fluid in a circular duct. The power law index for each case was then determined using Equation (3),

(3) $$\begin{array}{r} v_{normalized}=(\frac{3n+1}{n+1})\;v_{averageN}\;(1-{\left(y_{i}^{*}\right)}^{\frac{n+1}{n}})+v_{0N} \end{array}$$

where v_normalized_ is the experimental velocity normalized by average velocity, n is the power law index, v_averageN_ is the average velocity calculated for the fitting and v_0N_ is the slip velocity calculated for the fitting. MATLAB's curve fitting tool was used to calculate n and v_averageN_, and the Levenberg-Marquardt algorithm was selected in the non-linear least squares method. The value of n indicates whether the fluid is Newtonian or non-Newtonian, with *n* = 1 for a Newtonian fluid, *n* > 1 for a shear-thickening fluid, and *n* < 1 for a shear-thinning fluid.

Differential capillary viscometry, a technique which has been used to determine viscosity in microchannels, presents a method for deducing viscosity from flow, and a more detailed description can be found in Wunderlich and Bausch (52). By integrating the power law index with respect to shear rate for each case, the viscosity-shear rate curve can be recovered. The viscosity for each case is calculated using Equation (4),

(4) $$\begin{array}{r} n\left(\overset{∙}{\gamma }\right)=\frac{d\ln\mu }{d\;\ln\dot{\gamma }}+1 \end{array}$$

where $\overset{∙}{\gamma }$ is the shear rate. Once the viscosities are known, the power law relation is then used to calculate the consistency index, K, using a log-log relation for viscosity and shear rate. This fitting also gives an ideal power law index, which is used for the realistic cases. The power law equation is given by Equation (5),

(5) $$\begin{array}{r} \mu =K{\overset{∙}{\gamma }}^{n-1} \end{array}$$

where K is the consistency index. Even though polymers tend to be better characterized by other fitting models, a simplified version of the power law equation is used to estimate parameters in an attempt to minimize error introduced by the estimation of too many parameters.

### *In silico* Study

The CFD study made use of two different geometries to validate and observe flow behavior in myocardial tissue. An idealized channel was used to compare computational results with experimental data. Confocal microscopy images of rat myocardium were reconstructed to develop a model based on realistic representation of myocardial tissue. This was used to observe flow in a more complex setup and to investigate the impact of myocardial flow region.

### Idealized Channel

**Figure 2:**
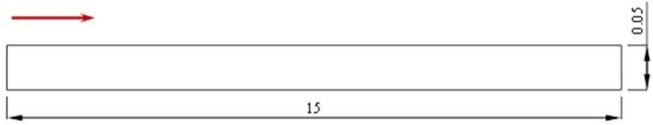
Idealized geometry used to simulate flow of fluid in microchannel.

An idealized channel was modeled and implemented in two-dimensions. The flow domain has a width of 50 μm and a length of 15 mm, as illustrated in Figure 2. These dimensions were chosen to match the dimensions of the microchannel used in the experimental study, and to enable validation of the computational study. Geometries were developed using DesignModeler and meshes were generated in ANSYS Meshing (ANSYS Inc., Lebanon, NH).

A grid independence study was conducted to determine the appropriate resolution level for the idealized simulations in this study. Grids of increasing refinement were defined, with element sizes of 6.25 × 10^−6^, 4.6875 × 10^−6^, 3.7594 × 10^−6^, and 3.125 × 10^−6^ m. The percentage error between the different meshes was found to be between 1 and 2%, and all results were computed on a mesh of element size 3.125 × 10^−6^ m. All the cells in the computational domain were the same size.

### Realistic Myocardial Tissue

**Figure 3:**
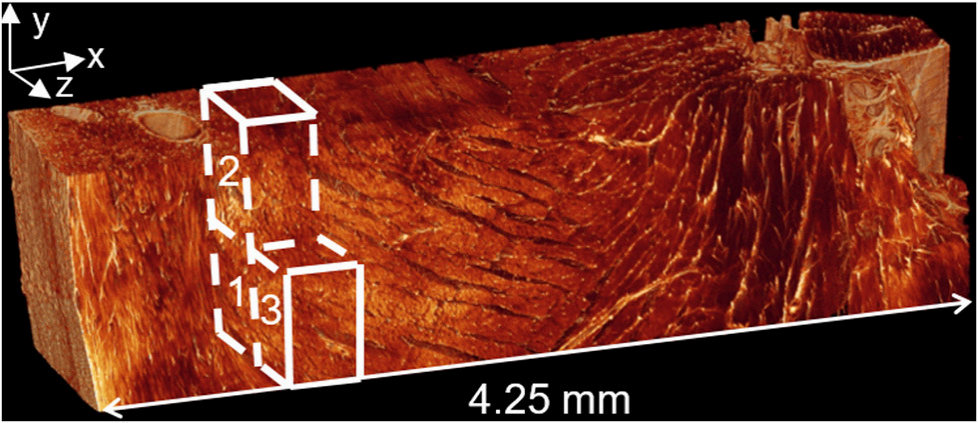
Confocal microscopy image of left ventricular tissue slice from a rat. Adjusted with permission from Sands et al. (58).

The realistic, three-dimensional geometry was obtained from confocal microscopy images of a rat's heart, illustrated in Figure 3 (58). Confocal microscopy is an optical imaging technique used for increasing optical resolution and contrast of a micrograph. It captures two-dimensional images at different depths of a sample and allows the reconstruction of these images into three dimensional structures. This geometry was used to investigate the manner in which three different myocardial flow regions, labeled 1, 2, and 3 in Figure 3, affect PEG dispersion in the tissue. The length of the tissue from endocardium to epicardium is ~4.25 mm.

**Figure 4:**
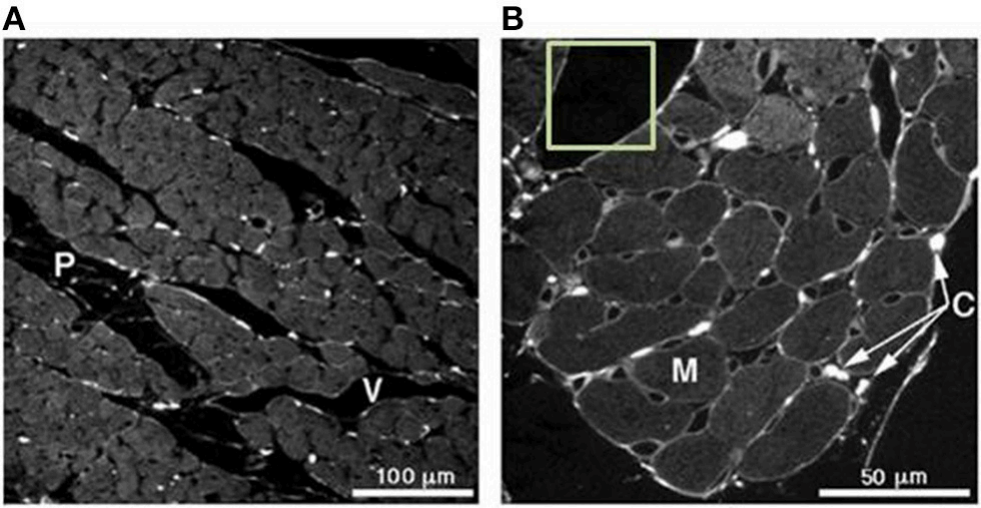
**(A)** Slice taken from a mid-wall region of a rat's left ventricle analyzed under a 25x magnification lens. (P) indicates the cleavage planes and (V) the vasculature structure. **(B)** 63x magnification of the same tissue in **(A)**. Here, the myocytes can be seen (M) and collagen chords in (C). Picrosirius red was used for the staining of collagen. Adjusted with permission from Young et al. (59).

Figure 4 illustrates a magnified view of heart tissue. Various regions of interest can be observed in the image. A slice taken from the mid-wall region of a rat's left ventricle is shown in Figure 4A. This image is captured under a 25x magnification lens. The region labeled (P) indicates a cleavage plane, which is the interstitial space located between and around cardiomyocyte bundles, while (V) illustrates the microvasculature structure. A 63x magnification of the same tissue is illustrated in Figure 4B. The myocytes are indicated by (M) and the collagen chords by (C). In the computational model developed, it is assumed that flow of the hydrogel takes places in the cleavage planes found in the tissue.

**Figure 5:**
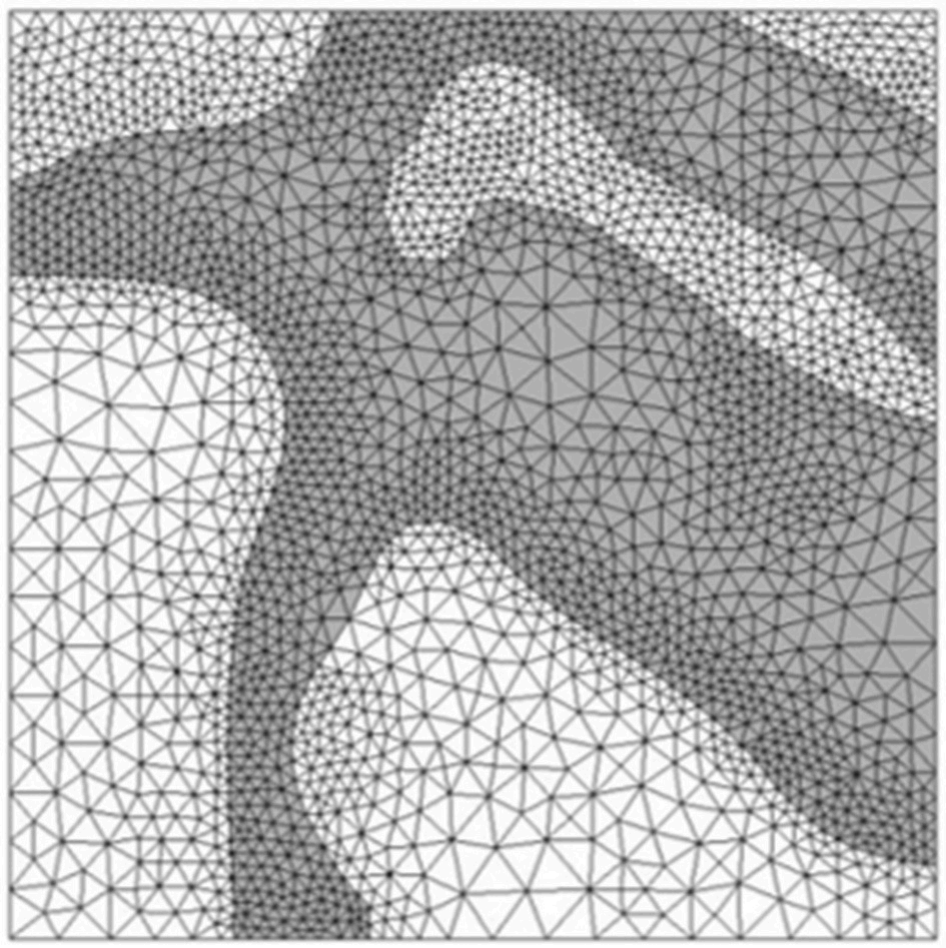
Tetrahedral mesh of heart tissue created in Simpleware. The gray zones represent areas where fluid can flow while white zones represent solid tissue.

Image reconstruction was carried out in Simpleware ScanIP (Simpleware, Exeter, UK). Stacks of high resolution and low resolution images were obtained from confocal microscopy. Using a thresholding technique, distinction was made between cleavage planes and solid myocardium, enabling reconstruction of the tissue block. Manual segmentation was employed and in some instances, myocardial material was removed to enable continuity between the cleavage planes. This was done to ensure that the fluid flow equations would not be violated. To examine the flow through the myocardial tissue, blocks of dimensions 660 × 560 × 440 μm were chosen as the region of interest, as illustrated in Figure 3. The blocks were taken as close to the epicardium as possible, where injection is likely to take place. Tetrahedral meshes of varying refinement were created using the Simpleware FE module. Figure 5 illustrates a sample mesh that was created. The gray zone represents the cleavage plane and is defined as a fluid region through which PEG can flow. The white region represents the tissue and is modeled as a rigid wall.

A grid independence study was carried out for the realistic geometry. In this instance, it was slightly harder to control element sizes as tetrahedral elements were used. The coarsest mesh had 274 477 cells, followed by a medium mesh of 425 136 cells and a fine mesh of 730 799 cells. The error between the medium and fine mesh was 1.5% and results were computed on the medium mesh.

### *In silico* Settings and Parameters

For all the above-mentioned cases, the Navier-Stokes equations were used to describe the flow of the fluid. The continuity equation is described by Equation (6) and the momentum equation by Equation (7),

(6) $$\begin{array}{r} \frac{\partial \rho }{\partial t}+\nabla \left(\rho \text{v}\right)=0 \end{array}$$

(7) $$\begin{array}{r} \rho \frac{\partial \text{v}}{\partial t}+\rho \text{v}.\nabla \text{v}+\nabla P=\mu \nabla^{2}\text{v} \end{array}$$

where ρ is density, **v** is the velocity vector, *t* is time, and *P* is pressure.

The computational frameworks were implemented in ANSYS Workbench 19.2 (ANSYS Inc., Lebanon, NH). Flow simulations were computed in FLUENT and CFD-Post was used for post-processing. PEG is modeled as a non-Newtonian, incompressible fluid with density ρ = 1,200 kg.m^−3^ (60). For viscosity, a non-Newtonian power law is used and is described in Equation (5). For this study, the gel is modeled in its most fluid state, prior to the start of gelation. In reality, PEG gel is a viscoelastic material with a non-Newtonian viscosity that changes as the solution gels. All the simulations were run under steady state conditions in order to match the conditions employed for obtaining the μPIV data. A mass flow rate was specified at the inlet boundary and a zero pressure condition at the outlet boundary. For the idealized geometries, slip was specified at the walls as the fluid in question is a polymer solution flowing in a small channel (53, 54). The slip for the realistic geometry could not be determined hence a no-slip condition was employed for those cases. With the exception of density, all other parameters were calculated from the experimental study and are presented in the results section. The viscosity model values and the mass flow inlet for the realistic tissue model are also calculated from experimental values and are presented in the results section. For most of the μPIV results, errors are minimized by normalization. For the purposes of capturing the varying flow rate in the CFD study, it was necessary to use the absolute μPIV variables.

SIMPLE algorithm was employed for pressure-velocity coupling. A least squares cell based scheme was used for the gradient, and both pressure and momentum were solved with a second-order scheme. An algebraic multigrid solver was used for calculations. In order to validate the computational results, comparisons were made with those obtained from the μPIV experiments.

## Results

In this section, the results of the μPIV and computational studies are presented. The comparison between experimental and computational results is presented in the computational results section.

### *In vitro* Study

**Figure 6:**
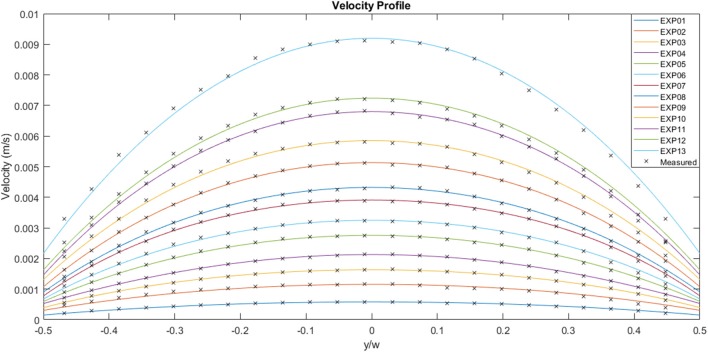
Experimentally measured flow velocities across the channel width for a range of flow rates (μPIV study). EXP01 is the lowest flow rate and EXP13 the highest.

Figure 6 illustrates the experimental curves following fitting of the original velocity data. The mass flow rate increases from case 01 to case 13, with case 01 reaching a maximum velocity of ~5.8 × 10^−4^ m.s^−1^ and case 13 exhibiting a maximum velocity of 9.2 × 10^−3^ m.s^−1^. The average velocity for case 01 is 4.4 × 10^−4^ m.s^−1^ and 6.9 × 10^−3^ m.s^−1^ for case 13. The ratio of the maximum to average velocity is ~0.75 for all the cases shown in the figure. The wall slip for each case, which also happens to be the minimum velocity, follows a similar trend to the other variables, with a value of 1.5 × 10^−4^ m.s^−1^ for case 01 and 2.2 × 10^−3^ m.s^−1^ for case 13.

**Figure 7:**
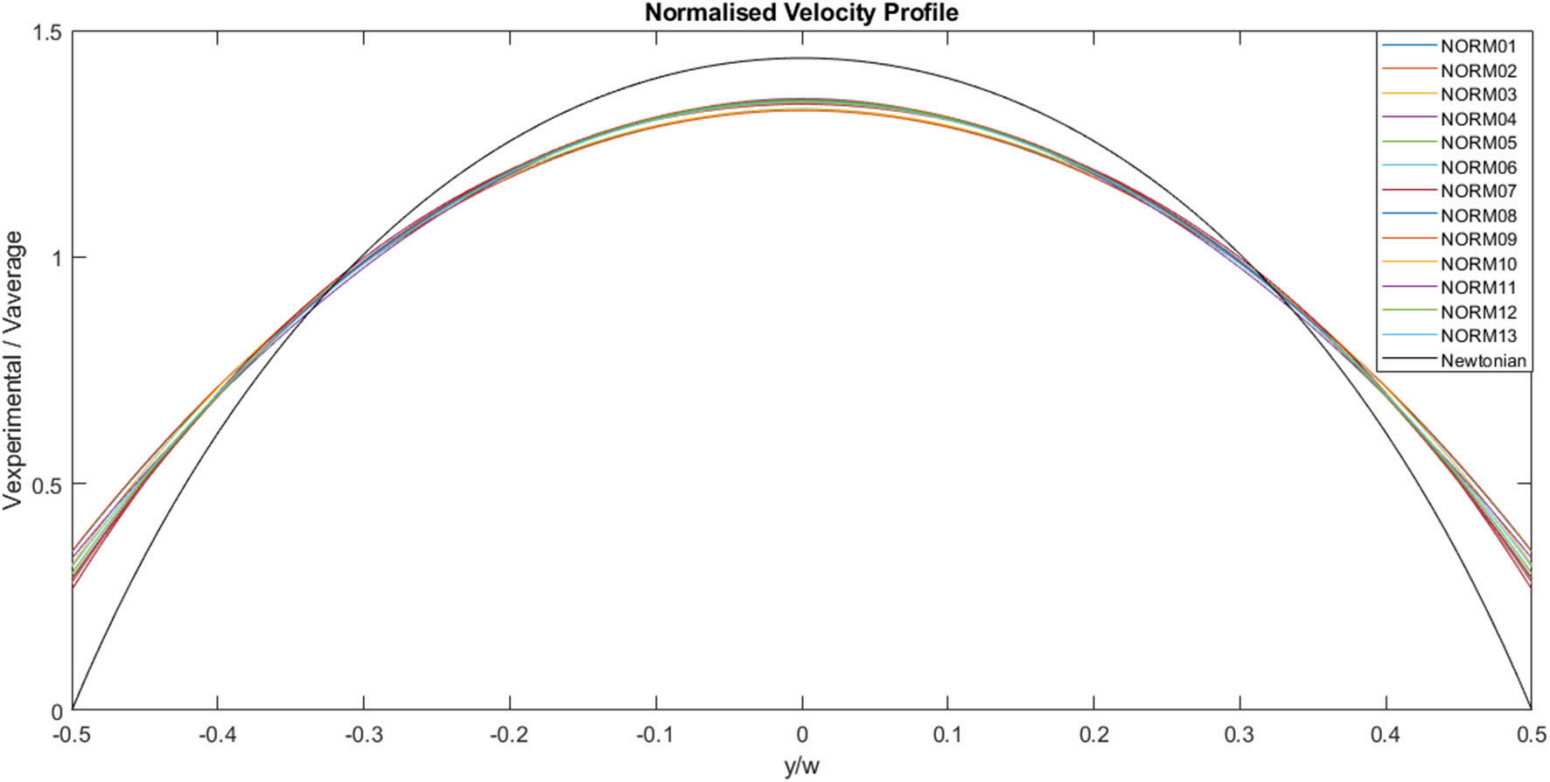
Normalized, experimentally derived, flow velocities across channel width for a range of flow rates. The solid black line represents the analytical profile for a Newtonian fluid.

The experimental curves were normalized using the appropriate average velocity for each case, as illustrated in Figure 7. The area under each curve is 1, the maximum value is ~1.3 and the minimum value is ~0.3. The solid black line represents the analytical Newtonian profile, and it is evident that the experimental curves are blunter than the analytical solution implying shear thinning behavior.

**Figure 8:**
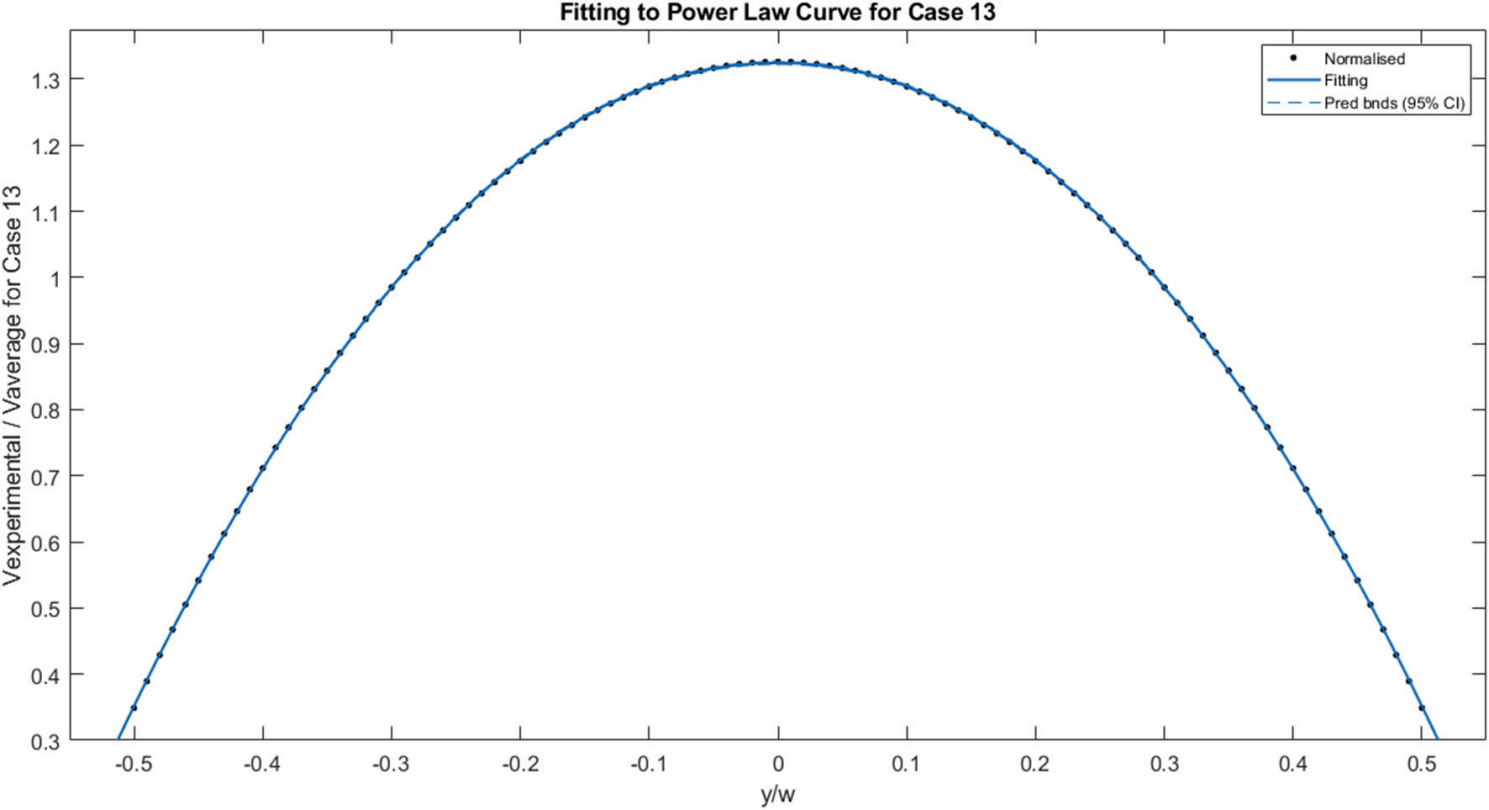
Fitting of normalized velocity profile data to the power law curve for Case 13.

Figure 8 illustrates the fitting of the normalized data to the power law curve. The example chosen for this specific image is Case 13 and the power law index, *n*, was 0.9389, indicating slight shear-thinning behavior. Other parameters were also calculated for the fitting, with v_averageN_ = 0.5198 and v_0N_ = 0.3186. Fitting was carried out for each case.

The calculated values obtained from the experimental data and the various fittings are detailed in Table 1. The shear rate ranges from 8 s^−1^ for Case 01 to 132 s^−1^ for Case 13. The flow rate ranges from 2.19 × 10^−8^ to 3.42 × 10^−7^ m^3^s^−1^. The power law index for most of the cases indicates shear-thinning behavior, and ranges from 0.793 to 0.943. Even with the anomaly presented by case 10, where the value indicates Newtonian/shear-thickening behavior, the fluid is assumed to be shear-thinning. Excluding case 10, viscosity varies from 0.434 to 0.887 kg.m^−1^s^−1^. In theory, the power law index remains constant for a given fluid. The power law indices for each of the flow rates are determined by fitting the normalized velocity data to the power law curve for a circular duct, on a case by case basis, as shown in Figure 8. Some variations are observed from case to case, and this is reflected by the slight variation in bluntness for each of the normalized velocity profiles, as illustrated in Figure 7.

**Table 1:** Shear rate, flow rate, power law index, and viscosity for each case.

**Case**	**$\overset{∙}{\gamma }$ (s^**−1**^)**	**$\overset{∙}{\text{q}}$ (m^**3**^s^**−1**^)**	***n***	**μ (kg.m^**−1**^s^**−1**^)**
01	8	2.19 × 10^−8^	0.943	0.887
02	17	4.35 × 10^−8^	0.928	0.816
03	24	6.14 × 10^−8^	0.860	0.642
04	30	7.86 × 10^−8^	1.04	1.15
05	39	1.02 × 10^−7^	0.905	0.705
06	47	1.21 × 10^−7^	0.850	0.561
07	56	1.46 × 10^−7^	0.793	0.434
08	62	1.60 × 10^−7^	0.877	0.602
09	73	1.90 × 10^−7^	0.874	0.583
10	84	2.18 × 10^−7^	0.885	0.600
11	97	2.52 × 10^−7^	0.880	0.578
12	104	2.68 × 10^−7^	0.918	0.683
13	132	3.42 × 10^−7^	0.939	0.742

Finally, the consistency index for the fluid was found to be 1.0 kg.s^n−2^.m^−1^ and the overall power law index of the fluid is 0.883. These two parameters were used for the viscosity model of the realistic tissue model, and the flow rate for case 13 was used as the inlet mass flow rate for the same model.

### *In silico* Study

**Figure 9:**
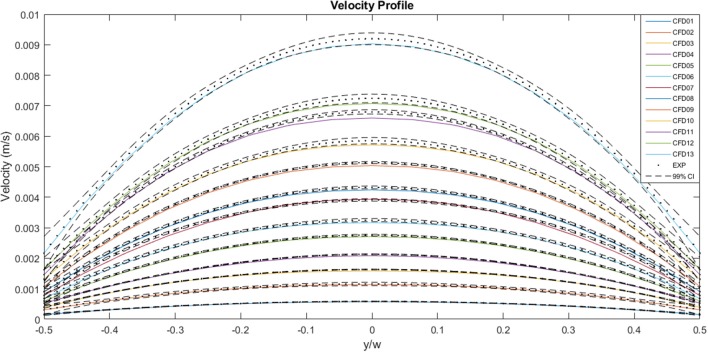
A comparison between the experimentally measured and computationally predicted flow velocities across the channel width at the mid-plane, for a range of flow rates. The solid lines represent CFD values, the dots represent experimental values and the dashed lines indicate the 99% confidence interval for those experimental values.

Figure 9 illustrates a comparison between the experimental and the CFD curves for the same parameters. The fluid property results presented in the *in vitro* study were applied to the computational model. The solid lines represent the CFD data, the dots represent the experimental values and the dashed lines represent the 99% confidence interval for those experimental values. Most of the CFD curves fit within the 99% confidence interval of the experimental values. Case 8 and 11 stand out as they have profiles that are significantly blunter than the experimental values, resulting in some of the values falling outside the 99% confidence interval. This discrepancy decreases with decreasing flow rate. Case 01, which has the lowest flow rate, has a maximum experimental velocity of 5.8 × 10^−4^ m.s^−1^ and a maximum CFD velocity of 5.7 × 10^−4^ m.s^−1^. The mean experimental and CFD values are 4.4 × 10^−4^ and 4.3 × 10^−4^ m.s^−1^, respectively. Case 13, which has the highest flow rate, has a maximum velocity of 9.2 × 10^−3^ m.s^−1^ for the experimental case, while the maximum CFD velocity is 9.0 × 10^−3^ m.s^−1^. The mean experimental value for the same case is 6.9 × 10^−3^ m.s^−1^ and the mean CFD value is 6.7 × 10^−3^ m.s^−1^.

**Figure 10:**
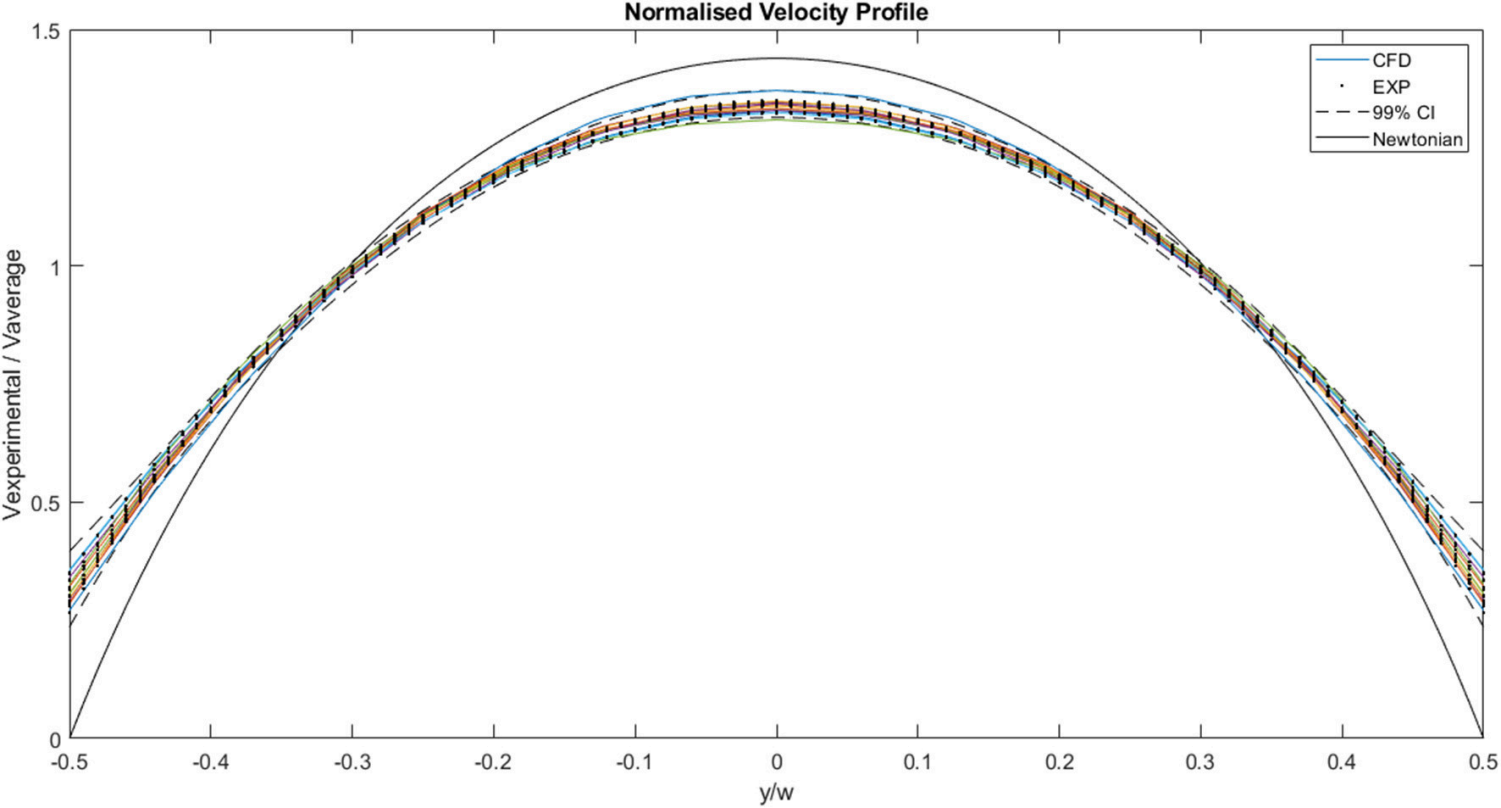
Comparison of normalized experimental and two-dimensional CFD data. The solid lines represent CFD values, the dots represent experimental values, the dashed lines indicate the 99% confidence interval for those experimental values and the black solid line represents the analytical, Newtonian solution.

A plot of the normalized values for the CFD and experimental curves is illustrated in Figure 10. The curves all follow a similar trend, and all the values fall within the normalized 99% confidence interval of Case 13. The area under each curve is 1. The maximum normalized velocity values for the CFD plots lie between 1.31 and 1.37. The minimum normalized velocity values, which correspond to the slip velocity, range from 0.27 to 0.35, for the CFD plots. All the profiles are blunter than the Newtonian profile, indicating shear-thinning behavior.

**Figure 11:**
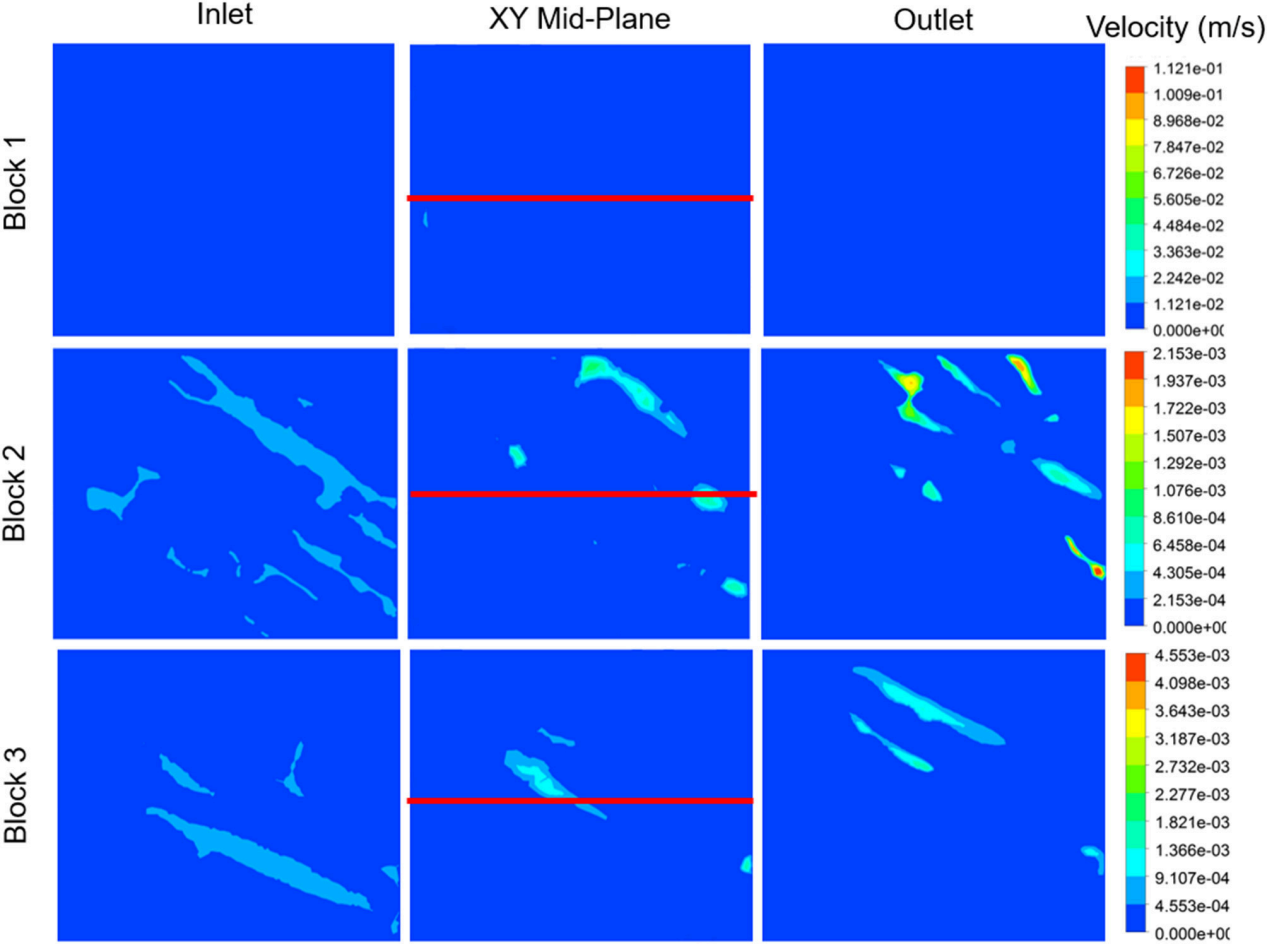
Velocity contour plots for each myocardial flow region, labeled Block 1, Block 2, and Block 3. The block names correspond to the sections illustrated in Figure 4. Velocity contours are plotted at the inlet, mid-plane, and outlet of each block. The red line indicates the centerline through the XY mid-plane, for which the velocity, shear rate, and viscosity are illustrated in Figure 12.

The non-Newtonian flow properties for the overall fluid were applied to different myocardial flow regions, as illustrated in Figure 11. It is clear, from the illustration, that the myocardial flow region has an impact on the manner in which the fluid passes through the tissue. Sections with slightly wider cleavage planes, such as Block 2 and Block 3, tend to achieve lower maximum flow velocities. The maximum velocity achieved for Block 2 is 2.15 × 10^−3^ m.s^−1^, and this is recorded on the outlet plane. Block 3, which is also has relatively wide cleavage planes achieves a maximum velocity of 4.55 × 10^−3^ m.s^−1^, however this value is not visualized on any of the planes illustrated in the diagram. The maximum value for block 1 is much higher than those measured on the selected planes. As such, the inlet, mid-plane, and outlet appear to have very little activity. The maximum flow velocity for Block 1 is 1.21 × 10^−1^ m.s^−1^ and this value is not visualized on any of the selected planes. This value is approximately two orders of magnitude higher than the maximum values for Blocks 2 and 3.

**Figure 12:**
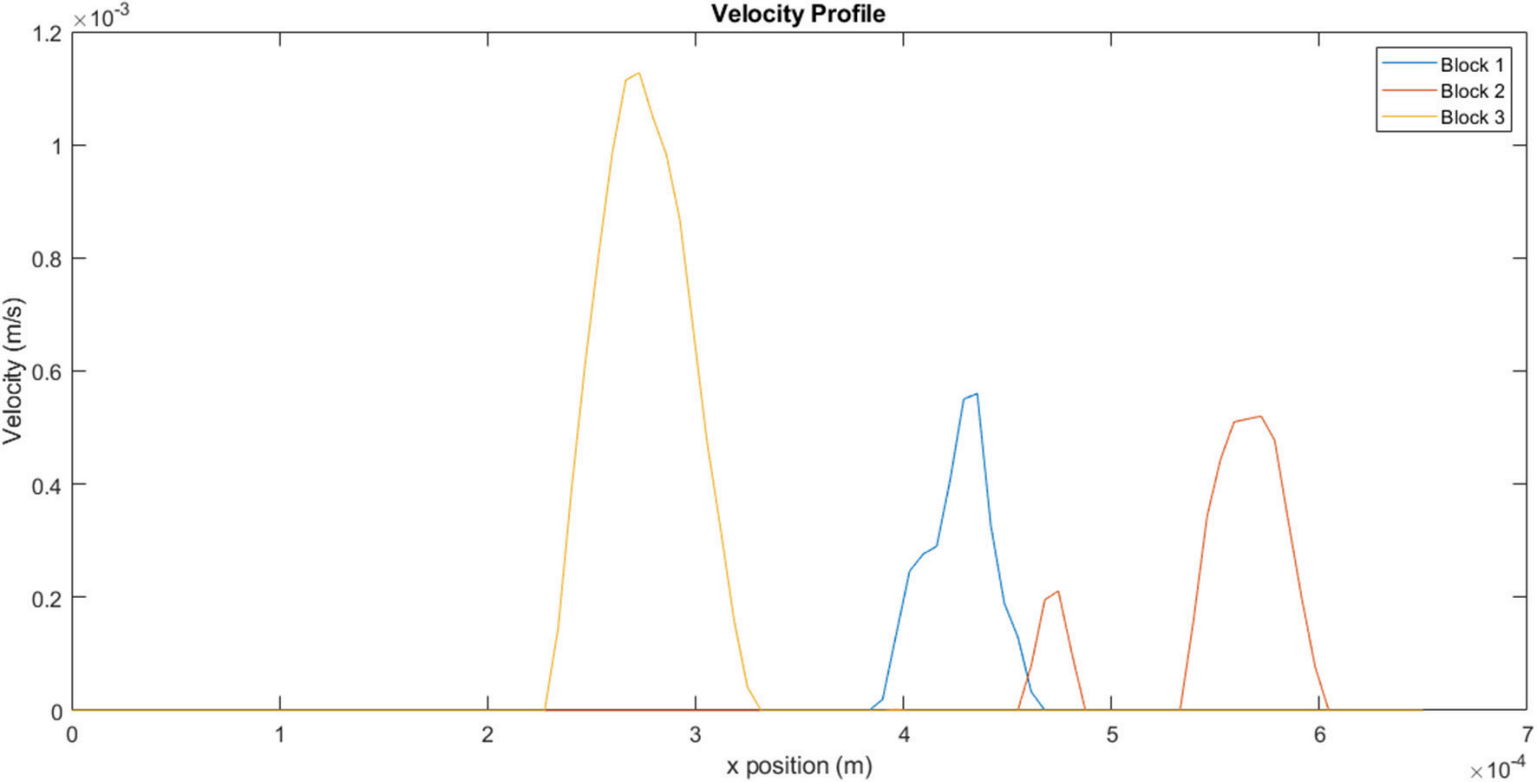
Velocity profiles for the different blocks taken from a line on the xy mid-plane, as indicated in Figure 11.

The velocity profiles along the xy mid-plane of each block, indicated by the red lines in Figure 11, are illustrated in Figure 12. The values reflected here are indicative of the flow velocities, shear rates, and viscosities experienced on the mid-plane, and may not necessarily reflect the flows experienced in the overall block. The same mass flow rate is specified at the inlet of each block. Even though the contour plots of Block 1 show very little activity in Figures 11, 12 clearly illustrates that the velocity values experienced on the mid-plane are fairly similar to those experienced on the mid-plane of Block 2. Block 1 has a maximum flow velocity of 5.60 × 10^−4^ m.s^−1^, while Block 2 has a peak flow velocity of 5.20 × 10^−4^ m.s^−1^. A slightly lower peak is also present for Block 2. Of the three blocks, Block 3 exhibits the highest peak, which has a value of 1.10 × 10^−4^ m.s^−1^.

**Figure 13:**
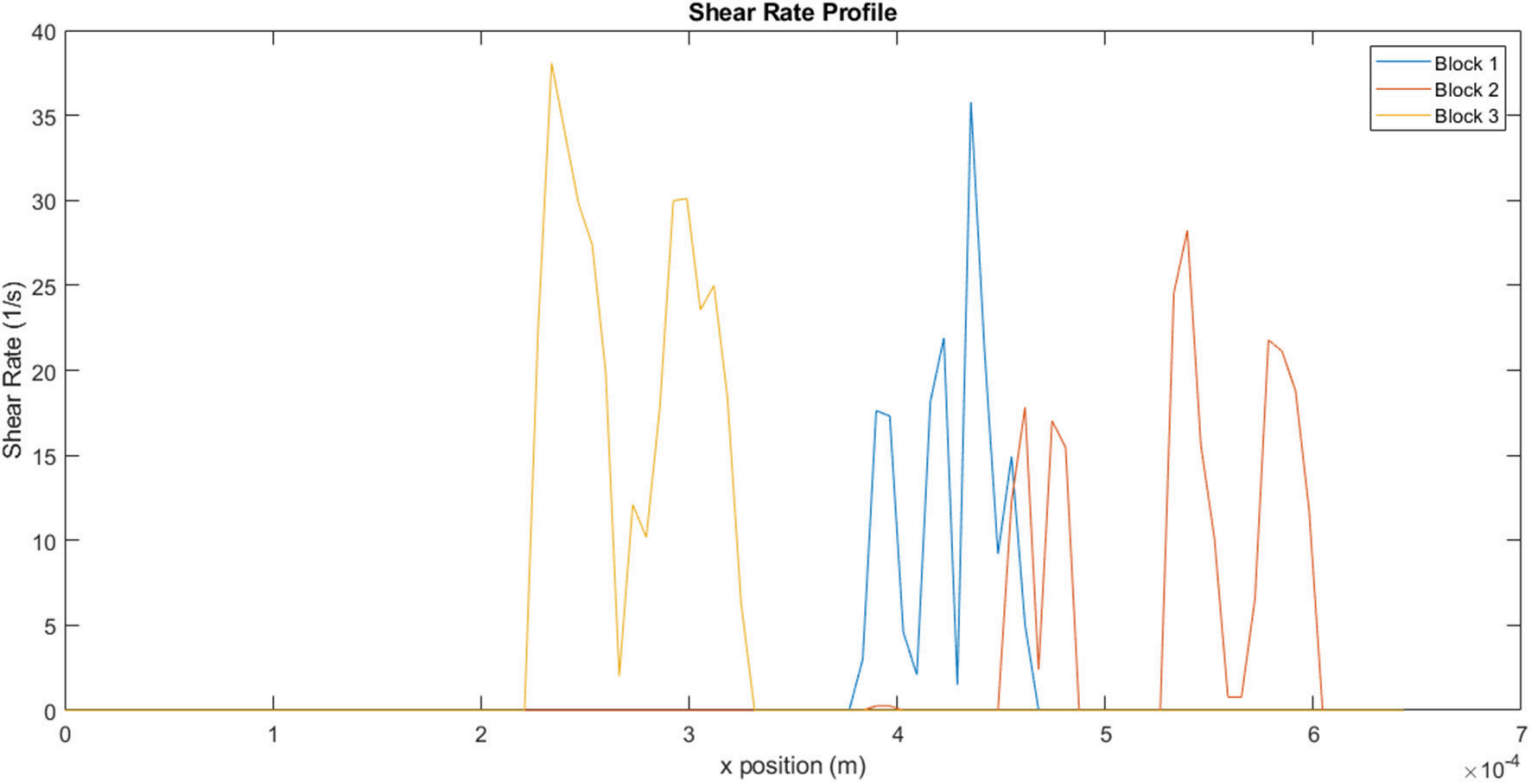
Variation in shear rate across the channels for the different myocardial flow regions.

Figure 13, which illustrates the shear rate within the channels, clearly demonstrates the range of conditions that the fluid would be exposed to within a single channel. The non-Newtonian behavior of the fluid becomes particularly important under these circumstances, as variation in shear rate would result in corresponding variations in viscosity. For a shear thinning-fluid, an increase in shear rate would result in a decrease in viscosity, thus decreasing the fluid's resistance to flow. For block 1, the fluid experiences a shear rate range of 1–36 s^−1^, within a single channel. The ranges for blocks 2 and 3 are 0.7–28 s^−1^ and 2–38 s^−1^, respectively. These results reflect the heterogeneity along a single line, on a single plane. Calculation of shear rates across the entire block would yield even greater variation.

## Discussion and Conclusions

Existing computational models of hydrogel injectates in myocardial tissue have examined injectates in the gelled state and have elucidated the impact of these gels on stresses in the myocardium (29–37). These studies have been useful in elucidating some of the mechanisms that contribute to the long-term efficacy of hydrogels. One of the main gaps in the literature is retention studies examining the manner in which fluids flow through myocardial tissue, prior to gelation. The work presented in this study makes a contribution toward understanding how fluids flow in myocardial tissue, and thereby how fluids are lost from the myocardium, and presents a framework for further studies of this nature. This complements observations such as those made by Van den Akker et al. who were able to visualize, in real-time, the wash out of stem cells via the venous circulation (61).

Dontula et al. who used 8 kDa PEG, found that PEG behaved as a Newtonian fluid up to 100 s^−1^ (42). Beyond this value, the viscosity of the solution was seen to increase, indicating shear-thickening behavior. Brikov et al. who used 4 kDa PEG, observed a similar phenomenon, although they found that the viscosity began increasing at a higher shear rate (47). These studies used standard, macroscopic, rheological techniques to reach their conclusions. In this study, there was interest in characterizing the gel on the microscale, where its behavior could deviate from macroscale observations. This is particularly true in the case of polymers, which experience restrictions on conformation in near-wall regions and behave slightly differently in microflows (53, 54). μPIV and CFD were used to examine the flow of PEG solution on the microscale, in channels resembling gaps found in heart tissue. A method presented in this paper, where the rheological parameters were derived from μPIV flow results, revealed that PEG solution behaves as a shear-thinning fluid on the microscale for shear rates between 8 and 129 s^−1^. These findings are in agreement with the work of Liu et al. who found that PEG was shear-thinning up until a shear rate of 2,000 s^−1^ (48). This was found to be true for PEG solutions of molecular weights ranging from 0.4 to 20 kDa. It is interesting to note that even lower molecular weight PEG solutions/gels exhibited different properties on the nanoscale to what was previously reported. There are various factors which may give rise to this Newtonian/non-Newtonian discrepancy seen in literature, including the testing conditions, molecular weight of PEG gel and the concentrations of PEG in the solutions. A more thorough investigation spanning spatial scales, testing methods, PEG molecular weights, and concentrations would be beneficial in determining how the material behaves for a range of different conditions.

The experimental results were useful not only for deducing the rheological properties of the fluid on the microscale, but also for providing data for input into the computational model. First, an idealized, two-dimensional CFD model was developed to enable meaningful comparison with experimental data, and for validation purposes. There was good agreement between absolute and normalized values, indicating that the methodology for deducing the rheological properties was effective. The computational framework was then implemented in realistic, confocal microscopy-derived microstructural geometry of rat myocardial tissue. This model was used to examine the effect of myocardial flow region on flow patterns within the tissue. It was evident, particularly from the shear rate results, that the fluid is exposed to a range of shear rates within individual channels, and this would lead to variation in viscosity values. Even though the channel widths were comparable for the different blocks, the distribution of shear rates within each channel is unique, hence the myocardial flow region does have an impact on fluid distribution. A number of *in vitro* studies have examined how different delivery routes affect retention (23). It was clear that the exact site of injection had an impact on fluid distribution. The channels in the sections explored in this study were of a comparable size, but even with that, differences in flow speed could be seen. Future studies could explore the flow of PEG gels in more complex microfluidic geometries with varying channel sizes mimicking the tissue microstructure (62). An abundance of narrow cleavage planes is likely to result in higher flow velocities and therefore, greater gel losses. Wider cleavage planes are likely to result in slow flow velocities, thereby encouraging more retention. This hypothesis could support the observations in a study by Kadner et al. where narrow channels characteristic of healthy tissue (and injection immediately after infarction) are likely to result in greater gel loss (24).

At this stage, there is no clarity regarding the exact shear rates that a hydrogel would be exposed to *in vivo*. It is highly likely that the shear rates will vary greatly over the cardiac cycle, and that some of values will be quite high. If PEG is indeed shear-thinning over a wide range of shear rates, the fluid would become less viscous as the shear rate increased, resulting in greater flow velocities and increased venous drainage. While this cannot be confirmed with the current model, the results presented here give clues as to why the retention of hydrogels has proven challenging to address. When the hydrogel is injected into the myocardium in a fairly liquid state, the solution is exposed to extensional flow as it moves through the narrow needle. Once the external pressure is removed and the gel moves through the myocardial tissue, the environment becomes far less predictable and the behavior of the solution prior to gelation is then harder to understand. The complex myocardial environment is likely to expose the injectate to a wide range of shear rates and may result in changes in the fluid's behavior, particularly on the microscale, where viscous forces tend to be dominant. The characterization of PEG solution over a wider range of shear rates, on the microscale, would therefore prove beneficial. In addition, a more realistic wall model which can account for the elastic effects of myocardial tissue, along with oscillatory shear experiments of the gel, would yield a more accurate representation of flow. Finally, a model which can account for the gelation process would enable verification of the period when the greatest losses occur.

## Data Availability

The datasets generated for this study are available on request to the corresponding author.

## Author Contributions

MN contributed to the design of the work, acquisition, analysis, interpretation of data, drafting and critical revision of the manuscript. AP and SB contributed to the acquisition, analysis and interpretation of data and critical revision of the manuscript. JK, AL, JM, and LS contributed to the acquisition of data and to critical revision of the manuscript. DB contributed to the design of the work and to critical revision of the manuscript. ND and TF contributed to the conception and design of the work and to critical revision of the manuscript. All authors provide approval for publication and agree to be accountable for all aspects of the work.

### Conflict of Interest Statement

The authors declare that the research was conducted in the absence of any commercial or financial relationships that could be construed as a potential conflict of interest.
